# NAD metabolism: Role in senescence regulation and aging

**DOI:** 10.1111/acel.13920

**Published:** 2023-07-09

**Authors:** Claudia Christiano Silva Chini, Heidi Soares Cordeiro, Ngan Le Kim Tran, Eduardo Nunes Chini

**Affiliations:** ^1^ Metabolism and Molecular Nutrition Laboratory, Kogod Center on Aging, Department of Anesthesiology and Perioperative Medicine Mayo Clinic College of Medicine Rochester Minnesota USA; ^2^ Metabolism and Molecular Nutrition Laboratory, Kogod Center on Aging, Department of Anesthesiology and Perioperative Medicine Mayo Clinic College of Medicine Jacksonville Florida USA; ^3^ Center for Clinical and Translational Science and Mayo Clinic Graduate School of Biomedical Sciences Mayo Clinic Jacksonville Florida USA

**Keywords:** aging, NAD^+^ metabolism, nicotinamide adenine dinucleotide, SASP, senescence

## Abstract

The geroscience hypothesis proposes that addressing the biology of aging could directly prevent the onset or mitigate the severity of multiple chronic diseases. Understanding the interplay between key aspects of the biological hallmarks of aging is essential in delivering the promises of the geroscience hypothesis. Notably, the nucleotide nicotinamide adenine dinucleotide (NAD) interfaces with several biological hallmarks of aging, including cellular senescence, and changes in NAD metabolism have been shown to be involved in the aging process. The relationship between NAD metabolism and cellular senescence appears to be complex. On the one hand, the accumulation of DNA damage and mitochondrial dysfunction induced by low NAD^+^ can promote the development of senescence. On the other hand, the low NAD^+^ state that occurs during aging may inhibit SASP development as this secretory phenotype and the development of cellular senescence are both highly metabolically demanding. However, to date, the impact of NAD^+^ metabolism on the progression of the cellular senescence phenotype has not been fully characterized. Therefore, to explore the implications of NAD metabolism and NAD replacement therapies, it is essential to consider their interactions with other hallmarks of aging, including cellular senescence. We propose that a comprehensive understanding of the interplay between NAD boosting strategies and senolytic agents is necessary to advance the field.

## Graphical abstract

The biological impact of NAD boosting on aging and cellular senescence is not completely understood. It appears that NAD is necessary for senescent cell function. Therefore, NAD boosting may increase senescence‐induced dysfunction. Combination of NAD boosters with senolytic therapy may be a promising approach to improve NAD‐targeted interventions, potentially benefiting age‐related diseases. However, no studies on this combination therapy have been reported yet.

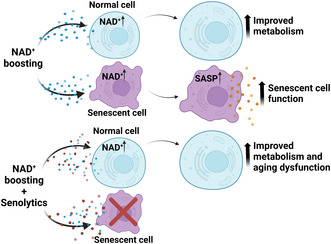

**Abbreviations**
NAD: (nicotinamide adenine dinucleotide)
KO: (knockout)
CD38: (cluster of differentiation 38)
CD73: (cluster of differentiation 73)
CD157: (cluster of differentiation 157)
SIRT: (silent mating type information regulation)
TRP: (tryptophan)
KYN: (kynurenine)
BST1: (bone marrow stromal cell antigen 1)
SASP: (senescence‐associated secretory phenotype)
COVID‐19: (coronavirus disease‐19)
AFAR: (American Federation of Aging Research)
NAD+: (oxidized nicotinamide adenine dinucleotide)
NADH: (reduced nicotinamide adenine dinucleotide)
ACMS: (2‐amino‐3‐carboxymuconic acid semialdehyde)
ENT: (equilibrative nucleoside transporter)
NA: (nicotinic Acid)
NAAD: (Nicotinic acid Adenine Dinucleotide)
NAM: (Nicotinamide)
NAMN: (nicotinic acid mononucleotide)
NAMPT: (nicotinamide phosphoribosyl transferase)
NMN: (nicotinamide mononucleotide)
NMNAT: (nicotinamide mononucleotide adenylyl transferase)
NR: (nicotinamide riboside)
NRK: (nicotinamide ribose kinase)
PARP: (poly‐adenoside diphospho‐ribose)
SARM1: (sterile alpha and TIR‐motif‐containing protein 1)
Slc12a8: (solute carrier family 12 member 8)
QA: (quinolinic acid)
QPRT: (quinolinate phosphoribosyl transferase)
NADS: (NAD synthase)
ACMSD: (alpha‐amino‐beta‐carboxymuconate‐epsilon‐semialdehyde‐decarboxylase)
ADPR: (adenosine diphospho‐ribose)
cADPR: (cyclic‐adenosine‐diphospho‐ribose)
ATP: (adenosine tri‐phosphate)
TIR: (toll/interleukin‐1 receptor)
IDO: (Indoleamine 2,3‐dioxygenase 1)
PGC1: (peroxisome proliferator‐activated receptor gamma co‐activator)
PnCA: (nicotinamidase)
CVD: (cardiovascular disease)
ECM: (extracellular matrix)
SC: (senescence cells)
MSCs: (mesenchymal stem cells)
AMPK: (5'‐adenosine monophosphate‐activated kinase)
HMGA: (high mobility group‐A protein)
SOD2: (superoxide dismutase‐2)
CCFs: (cytoplasmic chromatin fragments)
L1: (Line‐1)
ROS: (reactive oxygen species)
STING: (stimulator of interferon genes)
IRF3: (interferon regulator factor 3)
NF‐kB: (nuclear factor kappa‐B)
cGAMP: (cyclic guanidine monophosphate‐adenosine monophosphate)
c‐GAS: (cyclic GMP‐AMP synthase)
EXOs: (exosomes)
DDR: (DNA‐damage response)
DC: (dyskeratosis congenita).

## 1 INTRODUCTION

Aging is the most significant risk factor for most diseases, including diabetes, cancer, cardiovascular diseases, neurodegenerative disorders, and complications after infections such as COVID‐19. According to the American Federation of Aging Research (AFAR), the geroscience hypothesis proposes that “since aging physiology plays a major role in many—if not all—chronic diseases, therapeutically addressing aging physiology will directly prevent the onset or mitigate the severity of multiple chronic diseases” (https://www.afar.org/what‐is‐geroscience). Therefore, it is imperative to understand the mechanisms that govern the biology of aging and to identify strategies to promote healthier aging. However, although we have learned much about the biological processes that drive aging, no therapies are currently aimed at the basic biological hallmarks of aging, and we continue to treat diseases of aging in isolation.

In 2013, a land‐mark paper proposed the existence of nine biological hallmarks of mammalian aging, including genomic instability, telomere attrition, epigenetic alterations, loss of proteostasis, deregulated nutrient‐sensing, mitochondrial dysfunction, altered intercellular communication, stem cell exhaustion, and cellular senescence (López‐Otín et al., 2013). Recently, this list has been updated, proposing three new hallmarks of aging: disabled macroautophagy, chronic inflammation, and dysbiosis. These hallmarks of aging are comprehensively discussed in a 2023 review by López‐Otín et al. (2023). Interestingly, the nucleotide nicotinamide adenine dinucleotide (NAD) appears to interface with several of the biological hallmarks of aging, and changes in NAD metabolism have been proposed to be involved in the aging process (Chini et al., 2021; Johnson & Imai, 2018; Lautrup et al., 2019). Therefore, this review aims to discuss the role of NAD metabolism in aging, with a particular focus on the interactions between NAD metabolism and known hallmarks of aging, such as cellular senescence.

## 2 NAD METABOLISM IN AGING

NAD^+^ is an essential coenzyme in redox reactions and a substrate in non‐redox enzymatic processes such as cell signaling, DNA repair, and epigenetic modifications (Belenky et al., 2007; Saville et al., 2020; Yang & Sauve, 2016). NAD^+^ levels decrease with chronological aging in worms, flies, mice, and humans, appearing to be a conserved feature of aging across species (Braidy et al., 2011; Camacho‐Pereira et al., 2016; Chini et al., 2020; Covarrubias et al., 2020; Frederick et al., 2016; Gomes et al., 2013; Guest et al., 2014; Mouchiroud et al., 2013; Tarragó et al., 2018; Zhu et al., 2015). NAD^+^ levels also decrease in progeroid states and contribute to the metabolic dysfunction and decline in overall fitness observed during aging (Elhassan et al., 2019; Fang et al., 2019; Frederick et al., 2016; Gomes et al., 2013; Massudi et al., 2012; Tarragó et al., 2018; Yang et al., 2021). However, despite multiple studies showing an age‐related NAD decline in nearly every tissue in mice, the degree of NAD^+^ decline appears to vary between tissues and studies. For instance, the NAD^+^ decline in skeletal muscle of aged rodents has been reported to range anywhere from ~15%–65%, while in aged liver, most reports suggest a ~10%–50% decline (Camacho‐Pereira et al., 2016; Dall et al., 2018, 2019; Frederick et al., 2016; Gomes et al., 2013; McReynolds et al., 2021; Mouchiroud et al., 2013). In humans, a decrease in NAD^+^ levels was seen in the skin, brain, heart, monocyte‐derived macrophages, and liver by at least 10%–50% over the course of adult aging and in age‐related diseases (Abdellatif, Trummer‐Herbst, et al., 2021; Bagga et al., 2020; Clement et al., 2019; Elhassan et al., 2019; Guest et al., 2014; Massudi et al., 2012; Minhas et al., 2019; Parker et al., 2020). Therefore, decreased NAD^+^ availability may be a contributing and modifiable factor during aging and disease states (Chini et al., 2017).

### 2.1 Complexity of NAD metabolism

The metabolism of NAD is a complex and dynamic process playing a crucial role in organismal health (Chini et al., 2021). The electron transfer reactions involving NAD result in the interconversion between its oxidized and reduced forms. These reactions are reversible, and the sum of NAD^+^ and NADH is maintained. NAD^+^/NADH ratios are influenced by the metabolic status of the organism (Kulkarni & Brookes, 2019; Viña et al., 2016; Ying, 2006). Alterations in this ratio play a crucial role in the regulation of cellular functions since NAD participates in many signal transductions and protein modification reactions (Denu, 2003; Saville et al., 2020; Yang & Sauve, 2006, 2016). Maintaining the total NAD pool requires a significant energy expenditure, mainly because some tissues have very high NAD turnover rates (Liu, Su, et al., 2018; Zeidler, Chini, et al., 2022). Overall, the complexity of NAD metabolism can be explained by its multiple synthetic routes and consuming enzymes, many of which function primarily in cell signaling, DNA repair, and energy metabolism regulation (Kincaid & Berger, 2020; Strømland et al., 2021).

**FIGURE 1:**
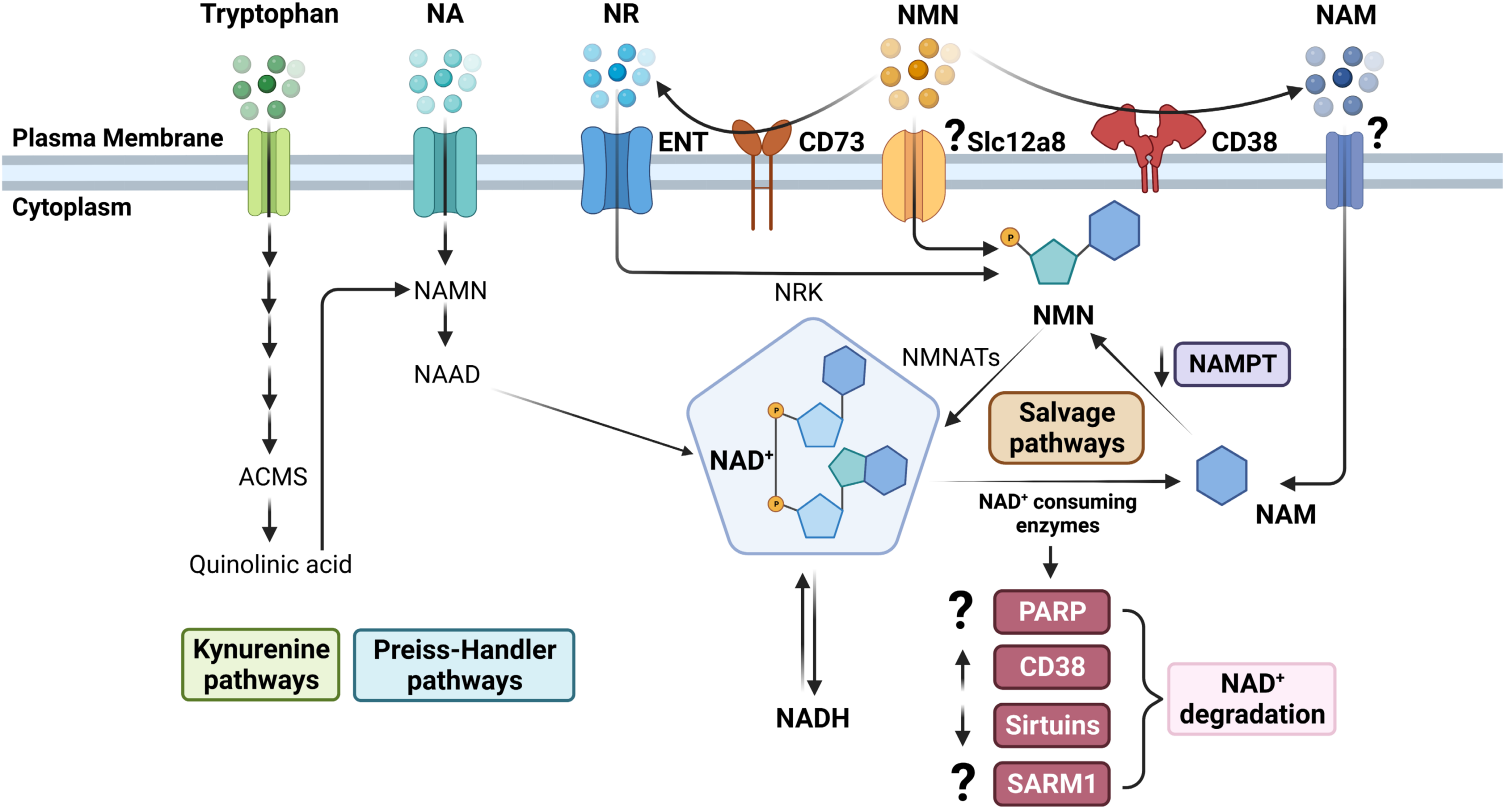
NAD metabolism pathways: regulation during aging. This figure summarizes the main NAD^+^ metabolism pathways, highlighting the entrance of NAD precursors in the cell, the synthesizing and degrading enzymes, and some of the changes that happens on these pathways during aging. From left to right: tryptophan enters the cells through neutral amino acids protein carriers, being used in the Kynurenine (de novo) pathway to produce NAD^+^; NA entrance is also mediated by a membrane carrier system, being used in the Preiss–Handler pathway to produce NAD^+^. NR enters the cell through ENTs, and NMN may use a specific Slc12a8 transporter. Both NR and NMN can be used in the salvage pathway to make NAD^+^. Outside of the cell, NMN can be converted to NR by CD73 or to NAM by CD38 before entering the cell. The NAM‐specific mechanism of uptake is still unknown. Decreased NAD^+^ levels with age appear to be driven by alterations in NAD metabolism enzymes. While levels of NAMPT and sirtuins have been shown to decrease during aging, levels of CD38 increase. PARP levels/activity have been shown to increase or decrease depending on the study, and the role of SARM1 in aging is still unknown. ACMS, 2‐amino‐3‐carboxymuconic acid semialdehyde; ENT, equilibrative nucleoside transporter; NA, nicotinic acid; NAAD, nicotinic acid adenine dinucleotide; NAD^+^, nicotinamide adenine dinucleotide; NADH, reduced nicotinamide adenosine dinucleotide; NAM, nicotinamide; NAMN, nicotinic acid mononucleotide; NAMPT, nicotinamide phosphoribosyltransferase; NMN, nicotinamide mononucleotide; NMNATs, nicotinamide mononucleotide adenylyl transferase; NR, nicotinamide riboside; NRK, nicotinamide riboside kinase; PARP, poly(ADP‐ribose) polymerase; SARM1, sterile alpha and TIR‐motif‐containing protein 1; Slc12a8, solute carrier family 12 member 8.

Levels of cellular NAD are intricately controlled by the balance between its synthesis and degradation (Figure 1). Synthesis of NAD in the body occurs through both extracellular and intracellular pathways (Cambronne & Kraus, 2020; Cantó et al., 2015; Liu, Su, et al., 2018). Multiple pathways for NAD synthesis have been described in mammalian cells and tissues, including the de novo pathway from tryptophan, the salvage pathway through the recycling or incorporation of nicotinamide, and the Preiss–Handler pathway (Figure 1). Importantly, the expression of different enzymes of synthesis and degradation of NAD vary at the subcellular, cellular, and tissue levels (Camacho‐Pereira et al., 2016; Cambronne & Kraus, 2020; Fortunato et al., 2022; Liu, Su, et al., 2018; Zeidler, Chini, et al., 2022).

The diet provides most precursors needed for NAD synthesis, including vitamin B3 precursors such as nicotinamide (NAM), nicotinic acid (NA), nicotinamide mononucleotide (NMN), nicotinamide riboside (NR), and tryptophan (Bogan & Brenner, 2008). The means by which these precursors enter the cell are still not completely elucidated. NR appears to enter the cell with the help of equilibrative nucleoside transporters (ENTs; Kropotov et al., 2021), while membrane carrier system mediates NA entrance (Said et al., 2007; Simanjuntak et al., 1990). A reduced form of NR (NRH), which has been shown to be more potent and faster than NR in increasing intracellular NAD^+^ levels (Giroud‐Gerbetant et al., 2019; Yang et al., 2020), also appears to enter the cell through ENTs (Giroud‐Gerbetant et al., 2019). The exact mechanism of cellular NAM uptake is still unknown, and the precise mechanism for the uptake of NMN is under debate. While some studies suggest that NMN is converted to NR before its cellular uptake (Kulikova et al., 2019; Ratajczak et al., 2016), others propose that it gets into the cell through a specific NMN transporter, Slc12a8, at least in the intestine (Grozio et al., 2019). Finally, tryptophan is taken into cells via carrier proteins that transport large neutral amino acids (Li, Yu, et al., 2022).

The de novo synthesis of NAD^+^ uses the essential amino acid tryptophan from the diet to generate quinolinic acid (QA) through the kynurenine pathway (Platten et al., 2019). The enzyme quinolinate phosphoribosyltransferase (QPRT) converts QA to nicotinic acid mononucleotide (NAMN), and then, nicotinamide mononucleotide adenyltransferase enzymes (NMNATs) catalyze the formation of nicotinic acid adenine dinucleotide (NAAD) from NAMN. In the final step, NAAD is converted into NAD^+^ by NAD synthetase (NADS) through amidation (Castro‐Portuguez & Sutphin, 2020). The de novo pathway appears to be limited to a few tissues, such as the liver, kidney, brain, and selected immune cells (Dale et al., 2000; Minhas et al., 2019; Wee et al., 2021). In fact, expression of de novo enzymes in mice is mainly restricted to the liver and kidney. A kinetic model of tryptophan metabolism predicts that although nearly 30% of tryptophan is converted to alpha‐amino‐beta‐carboxymuconate‐epsilon‐semialdehyde (ACMS) in the liver and kidney, only 0.03% of tryptophan is converted to ACMS in other tissues (Katsyuba et al., 2018; Palzer et al., 2018). Importantly, α‐amino‐β‐carboxymuconate‐ε‐semialdehyde‐decarboxylase (ACMSD), the enzyme that limits spontaneous cyclization of ACMS, has been shown to control cellular NAD^+^ levels and mitochondria function in mice (Katsyuba et al., 2018). In addition, it appears that the de novo pathway is crucial for the maintenance of kidney function, and its dysfunction has been implicated as a risk factor for the development of acute kidney injury in humans (Poyan Mehr et al., 2018; Späth et al., 2023).

In the salvage pathway, precursors such as nicotinamide (NAM) and nicotinic acid (NA) are used for the intracellular synthesis of NAD^+^ (Collins & Chaykin, 1972). Studies suggest that NAM is the main vitamin B3‐derived NAD^+^ precursor in mammals, and it also regulates NAD‐consuming enzymes by inhibiting NAD‐binding sites (Bitterman et al., 2002; Zeidler, Chini, et al., 2022). Nicotinamide phosphoribosyltransferase (NAMPT) and nicotinate phosphoribosyltransferase (NAPRT) are the two intracellular enzymes that catalyze the first step in the biosynthesis of NAD from NAM and NA, respectively (Hara et al., 2007; Revollo et al., 2004). The rate‐limiting enzyme NAMPT is expressed in all mammalian tissues (Audrito et al., 2020), and genetic deletion of this enzyme in mice is embryonically lethal (Revollo et al., 2007), suggesting that this route is essential in regenerating NAD. NAPRT can also be detected in most mouse and human tissues, but the expression and activity of this enzyme are higher in tissues such as the liver, intestine, heart, and kidney (Audrito et al., 2020; Duarte‐Pereira et al., 2016). These enzymes produce the intermediate precursors nicotinamide mononucleotide (NMN) and NAMN. NMNATs then convert NMN and NAMN into NAD^+^ or NAAD (Lau et al., 2009). Both NAMPT and NAPRT exist in intracellular and extracellular forms, and their expression increases in inflammatory conditions (Audrito et al., 2020; Gerner et al., 2018; Nacarelli et al., 2019; Nielsen et al., 2018; Revollo et al., 2007). NR is also utilized in the salvage pathway through its transformation into NMN by the nicotinamide riboside kinase (NRK1) (Bieganowski & Brenner, 2004). In the Preiss–Handler pathway, NAPRT converts dietary nicotinic acid (NA) into nicotinic acid mononucleotide (NAMN). NAMN is then converted into NAAD by NMNATs, and finally into NAD through amidation by the glutamine‐dependent NAD synthetase (NADS; Audrito et al., 2020; Lau et al., 2009). Some of these pathways appear to be more prevalent in certain tissues (Audrito et al., 2020), suggesting that the main pathway that regulates NAD synthesis may vary depending on the tissue.

Numerous non‐oxidative enzymes play roles in consuming NAD within cells and tissues. Some of the main NAD‐degrading enzymes are CD38, the poly (ADP‐ribose) polymerases (PARPs), sirtuins (SIRTs), and sterile alpha and TIR motif‐containing 1 (SARM1; Figure 1). The activity of these enzymes contributes to a decrease in NAD^+^ levels by breaking the glycosidic bond between the ribose of the dinucleotide and the nicotinamide ring. By hydrolyzing NAD^+^, these enzymes dictate NAD^+^ availability, thus regulating its levels and signaling function.

CD38 is now widely regarded as the primary NADase in mammalian tissues, and it plays a crucial role in regulating cellular NAD^+^ levels (Aksoy et al., 2006; Camacho‐Pereira et al., 2016; Escande et al., 2013; Tarragó et al., 2018). CD38 degrades NAD^+^ not only via its hydrolase activity, which results in the generation of NAM and ADP‐ribose (ADPR), but also via its ADP‐ribosyl cyclase activity, which generates small amounts of the calcium signaling molecule cyclic‐ADP‐ribose (cADPR; Howard et al., 1993). CD38 is expressed mostly on immune cells in response to cytokines, endotoxins, and interferon (Chini et al., 2019, 2020; Matalonga et al., 2017; Musso et al., 2001) and endothelial cells (Boslett et al., 2018; Chini et al., 2019; Zeidler, Chini, et al., 2022). Although CD38 can be found in the cytoplasm and intracellular organelle membranes, it is primarily found in the plasma membrane, with its catalytic site facing the outside of the cell. This is known as the CD38 “topological paradox” (Lee, Deng, & Zhao, 2022; Shrimp et al., 2014). While intracellular CD38 degrades intracellular NAD^+^, it appears that the ecto‐enzymatic activity of CD38 also modulates tissue NAD^+^ levels. This is likely mediated by the degradation of extracellular and circulating NMN and NAD^+^, regulating the availability of extracellular precursors to cells (Chini et al., 2020; Hogan et al., 2019; Shrimp et al., 2014). In support of this hypothesis, we have shown that inhibition of the CD38 ecto‐enzymatic activity both in vivo and in vitro increases levels of intracellular and tissue NAD^+^ and NMN and decreases NAM and ADPR levels (Chini et al., 2020; Ugamraj et al., 2022) Although one would expect that the NAM generated by CD38 could serve as a precursor for NAD through the salvage pathway, the net effect of CD38 ecto and endo enzymatic activities is a decrease in the NAD pool. Ultimately, the precise interplay between extra and intracellular CD38 and the dynamic interaction of the NAD pools remains to be completely defined.

We have also shown that dietary vitamin B3 partially regulates plasma NAM and NAM‐derived metabolites but not their tissue levels (Zeidler, Chini, et al., 2022). In contrast, tissue NAM is mainly generated by endogenous metabolism through CD38 NADase activity (Zeidler, Chini, et al., 2022). Importantly, CD38 has been shown to regulate many physiological processes and disease states, including metabolism, aging, obesity, diabetes, heart disease, asthma, and inflammation (Zeidler, Hogan, et al., 2022). BST‐1 (bone marrow stromal cell antigen 1, CD157), a paralog of CD38, was shown to degrade NR into NAM both in vitro and in vivo, and it is necessary for the conversion of orally administered NR into NAD^+^ (Yaku et al., 2021). BST‐1 expression appears to be more restricted than CD38, since it is mostly expressed in the intestine, kidney, and some immune cells (Quarona et al., 2013). The relevance of BST‐1 to disease states has not been extensively explored, but it has been reported that BST1 KO mice exhibit social deficits and anxiety‐like behaviors, which are rescued by the administration of NR (Gerasimenko et al., 2020).

Another group of enzymes that utilize NAD as a substrate is the poly ADP‐ribose polymerase (PARP). PARPs regulate DNA damage repair, epigenetics, and gene expression. Seventeen isoforms of this enzyme have been characterized, with PARP1 being the most extensively studied (Ryu et al., 2015; Vida et al., 2017). During severe DNA damage and genomic instability, PARPs consume a significant amount of cellular NAD^+^, leading to exhaustion of NAD^+^ and ATP levels, disruption of metabolism, and cell death (Fouquerel & Sobol, 2014; Ryu et al., 2015; Wang et al., 2019). Inhibition of PARPs, particularly PARP1, increases cellular NAD^+^ levels and regulates metabolism, mostly in cancer cells (Bai et al., 2011; Hurtado‐Bagès et al., 2020). NAM is also a byproduct of the enzymatic reaction.

Sirtuins are involved in numerous biological processes, including cell survival, senescence, proliferation, apoptosis, DNA repair, and cell metabolism. Seven mammalian sirtuins, SIRT1‐7, have been characterized, and they are found localized in various cellular compartments (Michishita et al., 2005). These proteins use NAD as a substrate to deacetylate lysine residues of target proteins, forming NAM and 2′‐O‐acetyl‐ADP‐ribose as their byproducts (North & Verdin, 2004). However, sirtuins have a low binding affinity for NAD and are likely influenced by the effects of other NAD‐dependent enzymes on NAD levels (Imai & Guarente, 2016; Sauve, 2010).

The sterile alpha and toll/interleukin‐1 receptor motif‐containing 1 (SARM1) is an important NADase in the nervous system (Essuman et al., 2017). The TIR (Toll/interleukin receptor) domain is necessary for SARM1 activity, and its dimerization consumes NAD^+^ (Essuman et al., 2017; Summers et al., 2016). In neurons, the dimerized SARM1 degrades NAD^+^ to produce NAM, ADPR, and small amounts of cADPR, much like CD38 (Essuman et al., 2017). SARM1‐mediated NAD^+^ degradation in neurons is observed during axonal degeneration, both in vitro and in vivo (Essuman et al., 2017; Gerdts et al., 2015; Summers et al., 2016). The role that SARM1 plays in NAD^+^ depletion in other tissues remains uncertain.

### 2.2 Regulation of NAD metabolism during aging

Several mechanisms have been proposed to explain the decline in NAD^+^ seen in aging. One theory is that synthesis of NAD^+^ decreases during the aging process (Figure 1). For example, levels of NAMPT, the rate‐limiting enzyme of the NAD^+^ salvage pathway, have been shown to be reduced with age in tissues, including human skeletal muscle (de Guia et al., 2019; Jadeja et al., 2018; Koltai et al., 2010; Stein & Imai, 2014), as well as in circulation (Yoshida et al., 2019). Several factors have been implicated in the regulation of NAMPT levels during aging, including changes in circadian rhythm, inflammation, and micro‐RNA levels (Strømland et al., 2021). However, a decline in NAMPT during aging has not been observed in all studies (Camacho‐Pereira et al., 2016; Yoshino et al., 2011). A decrease in NAD synthesis during aging may also be explained by a reduction in levels of nicotinamide mononucleotide adenylyl transferase (NMNAT) isoforms, as reported in the oocytes of aged mice (Wu et al., 2019). Additionally, overexpression of NMNAT in *Drosophila* extended their lifespan by improving oxidative stress response and mitochondrial function (Liu, Liu, et al., 2018). More research is still needed to fully understand the role of NMNATs in aging.

The de novo pathway has also been proposed to be dysregulated in aging. Aged macrophages have suppressed QPRT expression and de novo NAD^+^ synthesis. This suppression decreases mitochondrial respiration and induces a proinflammatory shift in the activation state of aged macrophages, demonstrating a role for the de novo pathway as a regulator of macrophage function in aging (Minhas et al., 2019). In contrast, other studies proposed that aging is associated with the activation of the IDO1‐KYN‐AhR signaling pathway. Indoleamine 2,3‐dioxygenase 1 (IDO1), the enzyme that catabolizes L‐tryptophan (L‐Trp) into kynurenine (KYN) in the de novo pathway, is activated in chronic inflammatory states, during the aging process, and age‐related diseases (Salminen, 2022b). However, the significance of these changes in the kynurenine pathway to the regulation of NAD^+^ levels in tissues during aging is still not clear.

Another hypothesis to explain the NAD^+^ decline in aging is that NAD^+^ consumption increases during aging (Figure 1). This idea is strengthened by studies showing that levels and activity of the NADase CD38 increase during aging, leading to NAD^+^ decline, and that CD38 knockout mice have preserved NAD^+^ levels in tissues during chronological aging (Camacho‐Pereira et al., 2016; Chini et al., 2020; Covarrubias et al., 2020; Tarragó et al., 2018). Importantly, the CD38 inhibitor 78c improved the survival of progeroid mice, ameliorated metabolic, structural, and molecular features of aging, and increased healthspan and longevity in naturally aged male mice (Peclat et al., 2022; Tarragó et al., 2018). This hypothesis is also supported by data on metabolic fluxes performed in young and aged mice. Using isotope tracers, McReynolds et al. (2021) showed that NAD synthesis is maintained with age, pointing to increased consumption as the primary driver of NAD decline in aging.

In the case of PARP1, both pro‐aging and anti‐aging functions have been described. One premise is that as levels of DNA damage increase with aging, the DNA repair enzyme PARP1 consumes and depletes NAD^+^. In support of the pro‐aging hypothesis, PARP activity was shown to increase with age in mice and human male skin (Braidy et al., 2011; Massudi et al., 2012; Mouchiroud et al., 2013). PARP inhibition prevented NAD^+^ depletion and premature aging in a mouse model with defective DNA repair and boosted NAD^+^ levels, improving mitochondrial fitness and function and skeletal muscle fatigue (Gomes et al., 2013; Scheibye‐Knudsen et al., 2014). In contrast, PARP1 deficiency in mice was shown to accelerate aging and cause the precocious manifestation of spontaneous carcinogenesis (Piskunova et al., 2008), suggesting an anti‐aging function for PARP1. In support of this hypothesis, Li et al. (2017) showed that PARP1 activity was lower in old mice, and these mice had decreased response to DNA damage. NMN treatment restored the reduced PARP1 activity and DNA damage response in the old mice, indicating that these responses were dependent on NAD levels. The mechanisms accounting for these different effects of PARP1 in aging mice are still not clear.

Sirtuins have also been shown to play a role in the aging process, even though they have low turnover rates for NAD and most likely do not play a major role in age‐related NAD^+^ decline. SIRT3 was among the first genes identified to extend lifespan (Benigni et al., 2019; Kaeberlein et al., 1999). Mice lacking SIRT3 have significantly shortened lifespans (Benigni et al., 2019) and spontaneously develop aging‐related diseases. Deletion of SIRT3 has a deleterious effect on mitochondrial biogenesis and function, which plays an important role in several age‐related diseases (Cao, Zhao, et al., 2022; Hu & Wang, 2022). Increased SIRT6 activity also correlates with increased lifespan among species, a phenotype attributed to its effect on protection from DNA damage (Korotkov et al., 2021; Taylor et al., 2022; Tian et al., 2019). SIRT6 overexpression in mice leads to a reduction in frailty and lifespan extension in both males and females (Roichman et al., 2021), while SIRT6 deficiency results in premature aging phenotypes and metabolic defects (Li et al., 2020; Mostoslavsky et al., 2006). In the case of SIRT1, whole‐body overexpression improved metabolism in mice but did not impact longevity (Herranz et al., 2010). On the contrary, both male and female brain‐specific SIRT1‐overexpressing transgenic mice had significant lifespan extension (Satoh et al., 2013). Activation of the NAD^+^/SIRT1‐PGC‐1α axis has been shown to be dysregulated in neurodegenerative diseases, such as Huntington's disease (Lloret & Beal, 2019), amyotrophic lateral sclerosis (Herskovits et al., 2018; Kim et al., 2007), and in accelerated aging pathologies such as Cockayne syndrome and Xeroderma pigmentosum (Fang et al., 2014; Scheibye‐Knudsen et al., 2014).

Another important consideration when studying NAD metabolism is the microbiome–host interaction. Studies using stable isotope tracing and microbiota‐depleted mice showed multiple connections between the microbiome and the host that shape NAD metabolism in vivo. First, it was shown that bacteria‐mediated deamidation via the nicotinamidase PnCA contributes to the NAD boosting effect of oral NAM and NR supplementation through the generation of NA and NAR (Shats et al., 2020). Interestingly, it was demonstrated that circulating host NAM enters the gut lumen and supports microbial NA synthesis that can be further used systemically as a tissue NAD precursor (Chellappa et al., 2022). Since disruption in the microbiome (dysbiosis) is an important hallmark of aging, it is essential to determine whether manipulations in NAD metabolism can ameliorate the age‐related disfunction of the microbiome, improving age‐associated diseases. In addition, it is possible that age‐related dysbiosis could also have a profound systemic effect on the host NAD metabolism since gut bacteria controls multiple aspects of metabolism.

Despite all the advances in investigating the role of NAD metabolism in aging, much remains to be understood about the specific mechanisms that control NAD^+^ levels in different tissues during aging. Fundamental questions about the role of NAD^+^ decline in aging and age‐related diseases are still unexplored. Most surprisingly, it is unknown to what extent low NAD^+^ levels can drive aging phenotypes and whether it can be considered an emerging hallmark of aging. In addition, the interplays between NAD^+^ metabolism and the “classical” biological hallmarks of aging, such as cellular senescence, remain to be fully characterized (van der Rijt et al., 2020). These gaps in knowledge have been driven, at least in part, by the lack of reliable animal models of tissue NAD^+^ decline and recovery that can separate changes in NAD^+^ from the pleiotropic effects of old age. The main reason why the induction of an NAD^+^ deficiency state in mice has been difficult to accomplish is that wild‐type animals can satisfy their NAD^+^ needs by metabolizing tryptophan (Trp) to NAD via the kynurenine (de novo synthesis) pathway and therefore do not heavily depend on dietary niacin (Figure 1; Castro‐Portuguez & Sutphin, 2020). Understanding the role of NAD metabolism in the biology of the aging process will be crucial for designing strategies to promote healthy aging.

## 3 NAD METABOLISM IN AGE‐RELATED DISEASES

NAD metabolism dysfunction is common during aging and various age‐related diseases such as cardiovascular diseases, liver and kidney diseases, sarcopenia/myopathy, cancer, neurodegenerative diseases, and others (Chini et al., 2017). However, the underlying mechanisms leading to the disruption of NAD metabolism in many of these pathologies remain unclear. Below, we will discuss some of the age‐related diseases where dysregulation of NAD metabolism plays an important role.

### 3.1 Cardiovascular diseases

Cardiovascular diseases (CVDs) have significant alterations in NAD^+^ metabolism (Abdellatif, Sedej, & Kroemer, 2021; Campagna & Vignini, 2023). Intracellular NAD^+^ levels decrease in these diseases, promoting dysfunctions in energy metabolism that affect proper heart pump function (Jiao et al., 2022; Nollet et al., 2023). CVDs associated with NAD dysfunction include atherosclerotic and other vascular diseases, ischemic cardiomyopathy, diabetic cardiomyopathy, arrhythmogenic cardiomyopathies, pathological cardiac hypertrophy, dilated cardiomyopathy, and heart failure with preserved ejection fraction (Abdellatif, Sedej, & Kroemer, 2021). NAD dysfunction during CVD appears to affect endothelial cells. In these cells, the inflammation process increases CD38 expression, causing NAD depletion, and inhibits eNOS function, impairing nitric oxide generation (Boslett et al., 2018; Li et al., 2004; Reyes et al., 2015). This NAD^+^ decline leads to a decrease in SIRT1 activity, which further decreases NO levels and increases cellular oxidative stress, leading to premature senescence and apoptosis in endothelial cells (Mattagajasingh et al., 2007). PARP1 is also activated by oxidative stress and inflammation during CVD, causing further NAD^+^ decrease (Pacher et al., 2002). NAD‐boosting through the administration of NMN, CD38 inhibition, and PARP inhibition have all been shown to improve aspects of CVD (Agorrody et al., 2022; de Picciotto et al., 2016; Li, Deng, et al., 2022; Tarragó et al., 2018).

### 3.2 Cancer

Dysregulation of NAD metabolism is observed in several types of cancer (Chowdhry et al., 2019; Gao et al., 2023; Navas & Carnero, 2021; Yaku et al., 2018). Cancer cells reprogram their metabolism, increasing their capacity for glucose uptake and their rate of glycolysis, among other adaptations, and these metabolic changes require increased amounts of NAD^+^ (Pavlova et al., 2022). This shift in NAD metabolism is thought to confer advantages to cancer cells, such as increased resistance to oxidative stress and DNA damage, enhanced cell survival and proliferation, and altered immune response (Yaku et al., 2018; Zhang et al., 2017). Changes in the expression of NAD metabolism enzymes, such as NAMPT, NAPRT, PARPs, sirtuins, and CD38, have been observed in many tumor types (Akanksha et al., 2022; Chalkiadaki & Guarente, 2015; Chowdhry et al., 2019; Demarest et al., 2019; Garten et al., 2015; Navas & Carnero, 2021; Shi et al., 2021). Besides dysregulations in cancer itself, cancer treatments can also influence NAD metabolism through the induction of DNA damage (Banerjee et al., 2022). Several studies have investigated the potential of targeting NAD metabolism as a strategy to enhance cancer treatment efficacy or to prevent drug toxicity (Beltrà et al., 2023; Chini et al., 2014; Dellavedova et al., 2023; Margier et al., 2022; Sauriol et al., 2023; Yoo et al., 2021).

### 3.3 Metabolic disorders

Various studies have shown that dysregulation of NAD^+^ metabolism is closely linked to age‐related metabolic disorders and that insulin secretion is regulated by different NAD‐dependent mechanisms (Abdellatif, Sedej, & Kroemer, 2021; Okabe et al., 2019; Wang et al., 2023). In obesity and insulin resistance states, for example, levels of NAD^+^ decrease, leading to a disruption in metabolic homeostasis (Okabe et al., 2019; Yamaguchi & Yoshino, 2017). These decreased NAD^+^ levels are associated with impaired mitochondrial function, increased oxidative stress, and reduced insulin sensitivity (Okabe et al., 2019; Xie et al., 2020). Therapeutic approaches that increase NAD^+^ levels in obesity and insulin resistance states, including dietary interventions, supplementation with NAD^+^ precursors, and CD38 inhibitors, have been shown to improve metabolic function in preclinical studies (de Castro et al., 2020; Escalante‐Covarrubias et al., 2023; Escande et al., 2013; Tarragó et al., 2018).

### 3.4 Fibrosis

Fibrosis can affect any organ and is a common feature of many chronic diseases, including liver cirrhosis, pulmonary fibrosis, and kidney disease. This pathological condition is characterized by the excessive accumulation of extracellular matrix (ECM) proteins, such as collagen, in organs and tissues, leading to organ dysfunction and failure (Henderson et al., 2020). Recent studies show that many NAD metabolism enzymes, including NAMPT, sirtuins, PARPs, and CD38, play a key role in ECM remodeling and fibrosis (Huang et al., 2023; Shi et al., 2021; Wu et al., 2023; Xu et al., 2021; Zhen et al., 2021). CD38, for example, has been linked with the development of pulmonary, renal, and systemic sclerosis through its role in regulating the expression of proinflammatory cytokines, chemokines, and senescence (Cui et al., 2022; Shi et al., 2021; Tao et al., 2023). Moreover, the deletion of CD38 in a model of muscular dystrophy, the mdx mouse model, was shown to fully restore NAD^+^ levels in the heart, skeletal muscle, and in the diaphragm (de Zélicourt et al., 2022). Inhibition of CD38 reduced fibrosis and improved treadmill performance in these mice (de Zélicourt et al., 2022).

### 3.5 Neurodegenerative diseases

Aging is the main risk factor for most neurodegenerative diseases, including Alzheimer's, Parkinson's disease, Huntington's disease, and amyotrophic lateral sclerosis (Hou et al., 2019). While the exact cause of neurodegenerative diseases is not fully understood, growing evidence suggests that NAD^+^ metabolism plays a critical role in pathological axon degeneration and the development and progression of these diseases (Cao, Wang, & Yang, 2022; Figley & DiAntonio, 2020; Icso & Thompson, 2022). In addition, several accelerated aging diseases exhibit NAD depletion and neurodegeneration, including ataxia telangiectasia, xeroderma pigmentosum group A, and Cockayne syndrome (Lautrup et al., 2019). SARM1, NMNAT2, and CD38 are dysregulated in neurodegenerative diseases and have been proposed as potential therapeutic targets (Bosanac et al., 2021; Cheng et al., 2021; Langley et al., 2021; Roboon et al., 2021; Takaso et al., 2020).

### 3.6 Short telomere syndromes

One of the hallmarks of aging is telomere shortening (López‐Otín et al., 2023). Diseases characterized by telomeres attritions, such as telomere syndromes, include dyskeratosis congenita (DC), aplastic anemia, and idiopathic pulmonary fibrosis (Kam et al., 2021; Papiris et al., 2022; Shin et al., 2022). Emerging evidence indicates that short telomere‐induced DNA damage response (DDR) plays a crucial role in the cellular dysfunction in DC (Sun et al., 2020). Telomere shortening/dysfunction in both humans and mice interferes with NAD metabolism, causing CD38 NADase hyperactivation and NAD^+^ decline (Sun et al., 2020). CD38 hyperactivity limits NAD bioavailability for PARP and sirtuin enzymes. Decreased PARP and sirtuin activities aggravate telomere/genome damage and mitochondrial abnormalities, contributing to cellular senescence and telomeropathies (Amano et al., 2019; Sun et al., 2020). CD38 inhibition and/or supplementation with NAD precursors appears to alleviate the deregulation of NAD metabolism and restore downstream processes (Stock & Liu, 2021).

## 4 SENESCENCE IN AGING

Senescence is one of the important hallmarks of aging. Senescent cells (SC) are broadly characterized by cell cycle arrest, resistance to apoptosis, and the production of secreted factors known as the senescence‐associated secretory phenotype (SASP; He & Sharpless, 2017; Wiley & Campisi, 2021). The SASP includes a variety of extracellular modulators, including cytokines, chemokines, proteases, pro‐fibrotic growth factors, lipids, and exosomes (EXOs), and it is one of the major contributors to the chronic inflammation that happens in aging (Basisty et al., 2020; Yin et al., 2021). Accumulation of SC in tissues has been observed in model organisms and humans during chronological aging and in many diseases of aging. The excessive generation and insufficient elimination of SC by the immune system in tissues appear to contribute to the pathology of aging (Kale et al., 2020). SC show profound changes in the expression of cell surface proteins that influence their recognition by the immune system (Giannoula et al., 2023; Wang, Johmura, et al., 2022). In addition, through their SASP, they can attract immunosuppressive and proinflammatory cells to tissues, contributing to age‐related tissue dysfunction (Salminen, 2022a). Transplantation of SC into young animals is sufficient to promote systemic or tissue‐specific dysfunctions (Xu et al., 2017, 2018). Interestingly, after one single blood exchange, aged mouse blood induced cell and tissue senescence in young animals, suggesting that senescence spreads through the blood (Jeon et al., 2022). Still, senescence and SASP may also have beneficial effects, especially in young organisms, mediating tumor suppression, wound healing, and the reprogramming of cells toward a stem‐like phenotype (Di Micco et al., 2021; Huang et al., 2022).

**FIGURE 2:**
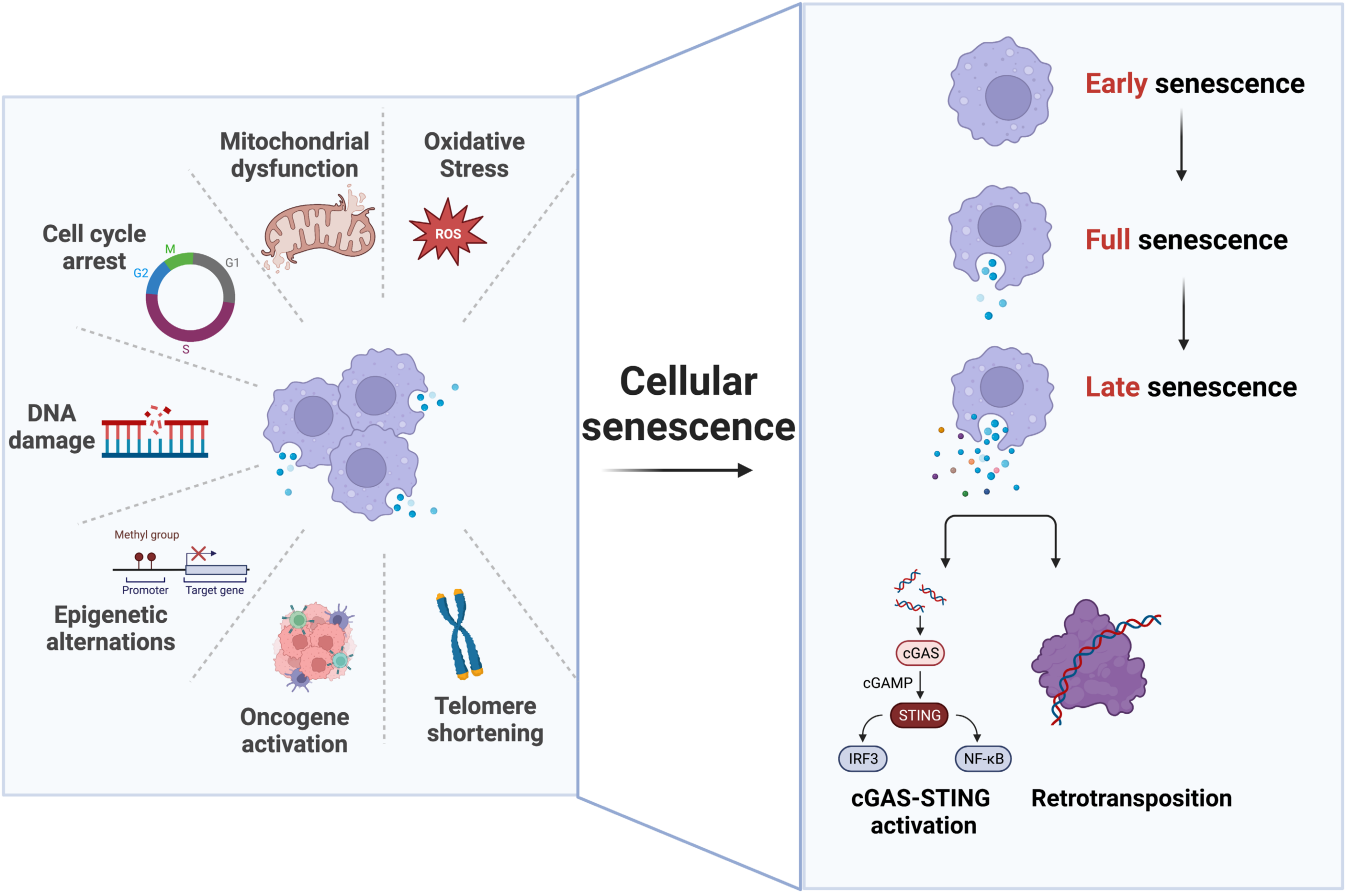
Development of cellular senescence. The left panel shows the main known inducers of cellular senescence. The right panel shows the progression of cellular senescence from early to late senescence. In late senescence, there is activation of the cGAS‐STING pathway alongside the retrotransposition and increased activation of the NF‐kB and interferon pathways. cGAMP, cyclic guanidine monophosphate‐adenosine monophosphate; c‐GAS, Cyclic GMP‐AMP synthase; IRF3, interferon regulator factor 3; NF‐kB, nuclear factor kappa‐light‐chain‐enhancer of activated B cells; ROS, reactive oxygen species; STING, stimulator of interferon genes.

Cellular senescence is characterized by a stable proliferative arrest triggered by multiple intrinsic stimuli and/or exogenous stress, including DNA damage, telomere attrition, oncogenic activation, mitochondria dysfunction, and oxidative stress (Wiley & Campisi, 2021; Figure 2). These signals intercept multiple effector pathways, converging on the activation of the cell cycle regulatory axes p53‐p21 and p16‐RB, which prevent the proliferation of damaged or stressed cells, usually in an irreversible fashion (Herranz & Gil, 2018). Other alterations of SC include epigenetic changes, such as enhanced activation of the regulatory histone H2AX, and nuclear adaptations, such as loss of lamin B1 (Crouch et al., 2022; Freund et al., 2012). Currently, it is accepted that there is not one single biomarker of SC, and that they can only be identified through the measurement of multiple markers (Robbins et al., 2021).

The cellular senescence phenotype is a dynamic process and may progress into a stage that is called “deep” or “late” senescence (Figure 2). This phenomenon is characterized by changes in SASP composition and a marked increase in the transcription of transposable elements, including LINE‐1 (L1) and others, which occurs much later after senescence onset (De Cecco et al., 2019; Gorbunova et al., 2021). These newly synthesized retrotransposon transcripts can engage in active transposition and accumulate in late‐senescent cell genomes (De Cecco et al., 2019). L1 activates a type‐I interferon (IFN‐I) response that is a marker of the late senescence stage and contributes to the maintenance of the SASP. Other processes driving continued change in SC are the extrusion of chromatin into the cytoplasm, resulting in the formation of cytoplasmic chromatin fragments (CCFs) and activation of the cGAS‐STING and NF‐kB signaling pathways that have crucial roles in the development of the SASP (Dou et al., 2017; Glück et al., 2017; Salminen et al., 2012; Schmitz et al., 2023; Zhu et al., 2021). In addition, the production of SASP by SC is also regulated by multiple signaling pathways, including p38 MAPK, TAK1, JAK2/STAT3, p53, mTOR, GATA4, Zscan4, and others (Freund et al., 2011; Laberge et al., 2015; Sun et al., 2022; Zhang et al., 2018).

Cellular metabolism plays a significant role in the regulation of the various signaling processes involved in cellular senescence. Like cancer cells, SC acquires a more glycolytic state, even in the presence of high oxygen levels. This metabolic shift leads to decreased NAD^+^/NADH ratios, increased AMP/ATP and ADP/ATP ratios, AMPK activation, and p53‐mediated growth arrest (Sabbatinelli et al., 2019; Wiley et al., 2016). Metabolic reprogramming is critical to senescent cell functions, including the production of SASP and modulation of immune responses within the tissue microenvironment (Soto‐Gamez et al., 2019). Several forms of metabolic stress can drive senescence and influence the SASP. Many drivers of mitochondria dysfunction have been shown to induce senescence, such as mtDNA depletion and mutations, inhibitors of the electron transport chain, loss of sirtuins SIRT3 and SIRT5, and disruptions of complex 1 assembly (Wiley et al., 2016; Wiley & Campisi, 2021). Lowering of the NAD^+^/NADH ratio and ATP depletion have been implicated in this mitochondrial dysfunction‐associated senescence (Wiley et al., 2016). Additionally, the loss of mitochondrial superoxide dismutase (SOD2) in mice drives cellular senescence (Velarde et al., 2012). There is increasing evidence that immune cells can also show signs of senescence. However, immunosenescence is even less characterized than senescence in fibroblasts due to the diversity of functions and phenotypes of different types of immune cells (Lee, Flores, et al., 2022). Interestingly, a senescent immune system was shown to drive systemic aging and senescence in solid organs (Yousefzadeh et al., 2021).

An increasing number of preclinical and early‐phase clinical studies of aging have implicated critical roles of senescence in a wide range of age‐associated disorders, including Alzheimer's disease, Parkinson's disease, atherosclerosis, chronic obstructive pulmonary disease, idiopathic pulmonary fibrosis, obesity/metabolic disease/insulin resistance, osteoporosis, and osteoarthritis (Khosla et al., 2022; Palmer et al., 2019; Schafer et al., 2018; Wissler Gerdes et al., 2020). This fueled the development of therapeutic agents that specifically target senescent cells. These drugs, named senotherapeutics, are classified into two major categories, senomorphics, and senolytics. While the senomorphics target senescent cells by modulating the expression or release of SASP components, the senolytics induce senescent cell death. Various clinical trials are underway based on extensive preclinical studies and small clinical trials demonstrating the benefits of senotherapeutics in multiple disease states. Preclinical studies and clinical trials on sonotherapeutics have been recently reviewed (Raffaele & Vinciguerra, 2022; Sun et al., 2022; Zhang et al., 2022).

## 5 NAD METABOLISM AND SENESCENCE

**FIGURE 3:**
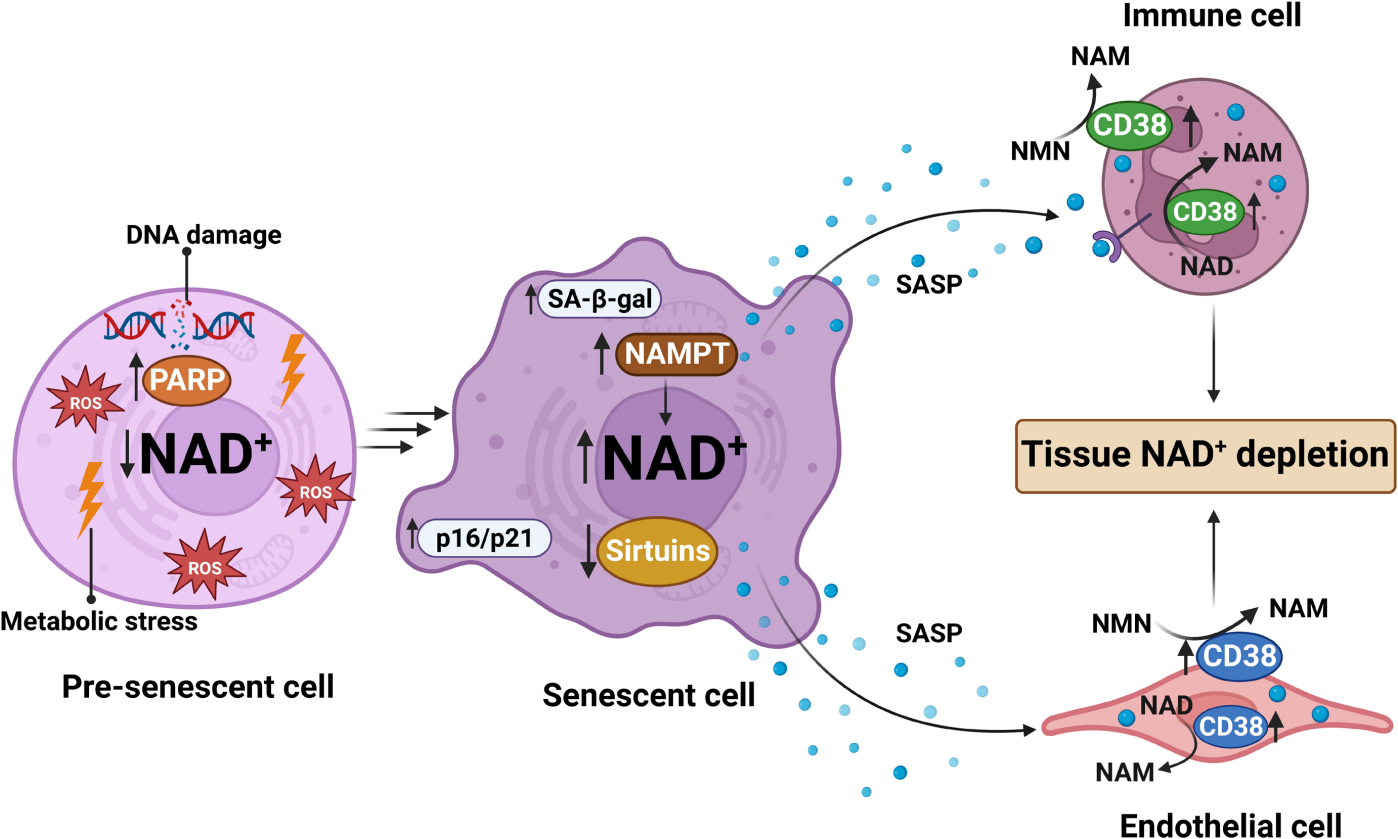
Interaction of cellular senescence and NAD metabolism in aging. Stimulus like metabolic stress and DNA damage induce PARP activation and NAD^+^ decline in cells, promoting cellular senescence. In senescent cells, NAMPT levels may increase, contributing to an increase in NAD levels that is necessary for SASP production. A decrease in sirtuins is also important for production of SASP‐associated secreted proteins. SASP released from senescent cells activate neighboring cells like immune and endothelial, increasing CD38 expression in these cells. CD38 as a NAD^+^ and NMN consuming enzyme causes overall tissue NAD^+^ depletion. NAD^+^, nicotinamide adenine dinucleotide; NAM, nicotinamide; NAMPT, nicotinamide phosphoribosyltransferase; NMN, nicotinamide mononucleotide; p16, tumor suppressor protein 16; p21, tumor suppressor protein 21; PARP, poly(ADP‐ribose) polymerase; ROS, reactive oxygen species; SASP, senescence‐associated secretory phenotype; SA‐β‐gal, senescence‐associated β‐galactosidase.

NAD^+^ homeostasis appears to have a complex role in the development and functions of SC (Figure 3). On the one hand, the accumulation of DNA damage and mitochondrial dysfunction induced by low NAD^+^ can promote the development of senescence (Desdín‐Micó et al., 2020; Han et al., 2016; Jadeja et al., 2018; Kim et al., 2021). On the other hand, the development of cellular senescence and its secretory phenotype appears to be highly metabolically demanding, and the low NAD^+^ state that occurs during aging may inhibit the development of cellular senescence, including the SASP (Nacarelli et al., 2019, 2020). However, to date, the impact of NAD^+^ metabolism on the progression of the cellular senescence phenotype has not been fully characterized.

It has been shown that SC have increased NAMPT expression and release extracellular NAMPT as part of their SASP (Kuehnemann et al., 2022). Importantly, a study investigating the role of NAMPT in SC demonstrated that NAMPT governs the proinflammatory SASP independent of senescence‐associated growth arrest (Nacarelli et al., 2019). The study proposes that the highly proinflammatory SASP that accompanies oncogene‐induced senescence is driven by HMGA‐mediated NAMPT expression and NAD^+^ metabolism, as well as DNA damage signaling. NAMPT promotes the proinflammatory SASP through NAD^+^‐mediated inhibition of AMPK kinase, suppressing the p53‐mediated inhibition of p38 MAPK to enhance NF‐κB activity. Moreover, supplementation with NMN enhanced the inflammatory environment and cancer progression in vivo (Nacarelli et al., 2019), showing that NAD^+^ metabolism regulates the strength of the proinflammatory SASP.

The levels and activity of sirtuins also regulate the development of senescence and SASP. SIRT1, for example, is downregulated by autophagy during senescence (Xu et al., 2020). A decrease in SIRT1 has been associated with multiple aspects of senescence, including SASP production, cell cycle arrest, stem cell senescence, and several senescence‐associated degenerative pathologies (Atkins et al., 2014; Chen et al., 2014, 2020; Hayakawa et al., 2015). SIRT6 has been shown to regulate multiple senescence‐associated biological processes, including oxidative stress, glucose and fat homeostasis, inflammatory responses, autophagy, genome integrity, and telomeres homeostasis (Li et al., 2021). SIRT6 deficiency induces endothelial and myocardial cellular senescence (Lee et al., 2020; Zhou et al., 2022), and SIRT6 knockout mice have premature aging and hyperinflammation (Mostoslavsky et al., 2006). Studies also show that SIRT3 is involved in the development of senescence. SIRT3 deletion accelerated ovarian aging in mice, as shown by a decrease in offspring sizes and increased expression of aging and inflammation‐related genes (Zhu et al., 2022). In addition, late passage mesenchymal stem cells (MSCs) display lower NAD^+^ content, reduced SIRT3 expression, and mitochondrial dysfunction. NMN supplementation in MSCs leads to a significant increase in intracellular NAD^+^ levels, NAD^+^/ NADH ratio, and SIRT3 expression, and ameliorates mitochondrial function and rescues the senescence phenotype (Wang, Sun, et al., 2022).

An important question in the field of NAD metabolism in aging is whether SC and their secretory phenotype can regulate NAD^+^ metabolism in non‐senescent cells. Recent studies, including two by our group (Chini et al., 2019, 2020; Covarrubias et al., 2020), indicate that SC can modulate NAD metabolism in other cells via regulation of the NAD^+^‐degrading ectoenzyme CD38. These studies show that immune cells are the primary cells that accumulate CD38 during aging and that senescent cells are, at least in part, responsible for the induction and recruitment of CD38^+^ cells to tissue during aging (Chini et al., 2020; Covarrubias et al., 2020). In addition, the SASP induced expression of CD38 in immune and non‐immune cells (Chini et al., 2019, 2020; Covarrubias et al., 2020). Depletion of SC or SASP in vivo decreased CD38 levels and partially recovered NAD^+^ levels in aging tissues (Chini et al., 2020), indicating that SC regulate the NAD^+^ decline during aging through CD38. A connection between CD38 and senescence was also demonstrated in a disease model of muscular dystrophy (mdx mice). Deletion of CD38 in mdx mice led to a fully restored heart function and structure, skeletal muscle performance improvements, and a reduction in inflammation and senescence markers (de Zélicourt et al., 2022).

## 6 NAD ^+^ REPLACEMENT (BOOSTING) THERAPY FOR AGING AND AGE‐RELATED DISEASES

Since NAD metabolism is dysregulated in aging and age‐related diseases, it has been proposed that restoring NAD^+^ levels may be a promising approach to promoting healthy aging. Oral supplementation of nicotinamide, NR, niacin, or other dietary precursors of NAD^+^ have been investigated as therapies to improve healthspan, longevity, and as potential treatments for multiple diseases states (Abdellatif, Trummer‐Herbst, et al., 2021; Hou et al., 2021; Miao et al., 2020; Okur et al., 2020; Pirinen et al., 2020; Poyan Mehr et al., 2018). However, many questions remain unanswered about the safety and efficacy of NAD^+^ “boosting” therapies for aging and age‐related diseases. The abundance of clinical trials of NAD^+^ “boosting” therapies in human subjects for conditions such as Alzheimer's disease, metabolic syndrome, heart conditions, and even for treatment of COVID‐19 infection/symptoms make it of increased relevance to understand the mechanisms by which these compounds regulate human physiology.

The clinical trials on NR and NMN administration demonstrated their safety regarding toxicity and their ability to effectively increase NAD levels in healthy volunteers. However, long‐term human safety trials evaluating the safety of NAD boosters such as NR and NMN are still lacking. The current data originate from relatively short‐term studies with a small number of participants, and it is not known if any of the NAD‐boosting strategies are superior to the others or if specific diseases would benefit from a particular NAD precursor intervention. Complications arise by the fact that the regulation of NAD metabolism is complex, and supplementation with NAD precursors such as NMN, NR, and NAM may cause distinct effects on downstream metabolites (Brakedal et al., 2022; Okabe et al., 2022; Reiten et al., 2021; Trammell et al., 2016). Also, these precursors differ in their mechanisms of absorption in the gut and how they enter the cell. Importantly, several studies with the NAD precursor nicotinamide riboside (NR) failed to demonstrate clinical or functional effects in the obese and the elderly (Dollerup et al., 2018, 2020; Jensen et al., 2022; Martens et al., 2018), highlighting the importance of large, double‐blind, randomized studies.

Although there is no evidence that treatment with NR or NMN for prolonged periods stimulated tumor development in animals, it is possible that during cancer progression and treatment, elevated NAD^+^ levels may have deleterious effects on cancer development by promoting cell survival, growth, inflammation, and increasing resistance to radio‐ and chemotherapy. In fact, increased NAD^+^ levels promoted inflammation and features of aging in some studies. For example, it was shown that NMN intake in mice increased NAD^+^ levels, influencing the secretory activity of senescent cells to promote cancer progression in vivo (Nacarelli et al., 2019). In contrast, several studies have described that NAD^+^ supplementation with NR reduces neuroinflammation and other inflammatory conditions (Doke et al., 2023; Hou et al., 2021; Stock et al., 2023), suggesting that the role and mechanism of action of these precursors in inflammatory diseases and aging is complex, and it is not fully understood.

Numerous studies have also shown that pharmacological inhibition of CD38 may be a potential therapeutic target for aging and metabolic diseases. Apigenin, a flavonoid that is an inhibitor of CD38, improved metabolic syndrome, renal injury, neuroinflammation, and osteoarthritis in animal models (Escande et al., 2013; Gil Alabarse et al., 2023; Ogura et al., 2020; Roboon et al., 2021). In addition, a specific thiazoloquin(az)olin(on)e CD38 inhibitor, 78c, prevented age‐related NAD^+^ decline and ameliorated physiological and metabolic dysfunctions such as glucose intolerance, muscle dysfunction, reduction in exercise capacity, and cardiac function in aging mice (Tarragó et al., 2018). Furthermore, 78c increased the lifespan and healthspan of naturally aged mice with a 10% increase in median survival, accompanied by improvements in exercise performance, endurance, and metabolic function (Peclat et al., 2022). 78c is a potent and specific inhibitor of CD38, and it is 10‐fold less potent against the cyclase activity than the hydrolase activity of CD38 (Tarragó et al., 2018). Antibodies that specifically block the CD38 NADase activity have been recently developed and shown to increase NAD^+^ levels in mice tissues and human cells (Ugamraj et al., 2022). Investigating the use of these antibodies in preclinical and human clinical trials will be essential to understand the role of CD38 as a pharmacological target for diseases of aging.

Tables 1 and 2 below summarize some of the preclinical and human studies that investigated the effect of different types of NAD‐boosting agents in specific age‐related pathological conditions.

**TABLE 1:** Preclinical studies.

Animal model	Intervention	Disease	Outcome	References
Tert^−/−^ mice	NR	Telomere disorder	Improved telomere integrity	Stock et al. (2023)
			Improved telomere‐dysfunction‐induced inflammation	
APP/PS1 mice	NR	Alzheimer	Reduced neuroinflammation, DNA damage, cognitive impairment, and improved dysbiosis	Hou et al. (2021)
Atm^−/−^ mice	NR	Neurodegeneration	Prevented neurodegeneration, suppressed senescence and neuroinflammation, and improved motor function	Yang et al. (2021)
Wistar rats	NR	Acute kidney injury	Did not ameliorate tubular damage nor the initiation of fibrosis	Morevati et al. (2021)
C57BL/6	NR/NMN	Cisplatin‐ and ischemia‐reperfusion‐induced kidney injury	Protected from kidney dysfunction, tubular injury, and apoptosis induced by cisplatin. Reduced kidney damage after ischemia‐reperfusion injury	Doke et al. (2023)
C57BL/6	NMN	Aging	Ameliorated age‐associated physiological decline in mice	Mills et al. (2016)
		Polycystic ovary syndrome (PCOS)	Normalized increased adiposity and hepatic lipid deposition	Aflatounian et al. (2022)
		Doxorubicin‐induced cardiotoxicity	Protected against toxicity in heart and skeletal muscle and improved loss of physical function	Margier et al. (2022)
		Chemotherapy‐induced cognitive impairment	Prevented cisplatin‐induced abnormalities, including cognitive function, without affecting the antitumor efficacy of cisplatin	Yoo et al. (2021)
		Metabolic disorder	Ameliorated metabolic disorders induced by a high‐fat diet	Zhang et al. (2023)
C57BL/6	NAM	Obesity	Improved hepatic steatosis, inflammation, and glucose tolerance	Mitchell et al. (2018)
		Severe malnutrition‐induced liver dysfunction	Improved hepatic steatosis and mitochondrial changes	Hu et al. (2022)
Swiss albino mice	Apigenin	Neuroinflammation	Neuroprotective effect against LPS‐induced neurotoxicity	Roboon et al. (2021)
C57BL/6	Apigenin	Obesity	Improved several aspects of glucose and lipid homeostasis	Escande et al. (2013)
C57BL/6	78c	Demyelination and regeneration	Increased oligodendrocytes and remyelinated axons after spinal cord demyelination	Langley et al. (2021)
		Aging	Increased lifespan and healthspan	Peclat et al. (2022), Tarragó et al. (2018)

**TABLE 2:** Human studies.

Group studied	Intervention	Treatment	Outcome	References
Twin pairs who were discordant for BMI	NR	5 months	Improved muscle mitochondrial number, myoblast differentiation, and gut microbiota composition independent of BMI	Lapatto et al. (2023)
Immunosuppressed solid‐organ transplant recipients	NAM	12 months	Did not lower the numbers of keratinocyte cancers or actinic keratoses	Allen et al. (2023)
Healthy middle‐aged	NMN	12 weeks	Arterial stiffness tended to decrease	Katayoshi et al. (2023)
Healthy middle‐aged adults	NMN	2 months	Safe and well tolerated	Yi et al. (2023)
			Increased walking distance and decreased blood biological age	
			No effect on insulin sensitivity	
Healthy aged men	NMN	6 and 12 weeks	Improved gait speed and performance in the left grip test	Igarashi et al. (2022)
			No significant effect on body composition	
Newly diagnosed Parkinson's disease patients	NR	1 month	Induced transcriptional upregulation of processes related to mitochondrial, lysosomal, and proteasomal function in blood cells and/or skeletal muscle	Brakedal et al. (2022)
			Decreased levels of inflammatory cytokines in serum and cerebrospinal fluid	
Mitochondrial myopathy patients	Niacin	10 months	Significant increases in muscle strength and mitochondrial biogenesis	Pirinen et al. (2020)
Cardiac surgery patients	NAM	Administered before cardiac surgery	Less acute kidney injury	Poyan Mehr et al. (2018)
Skin cancer high‐risk patients	NAM	12 months	Decreased incidence of non‐melanoma skin cancer	Chen et al. (2015)

## 7 CONCLUSION

**FIGURE 4:**
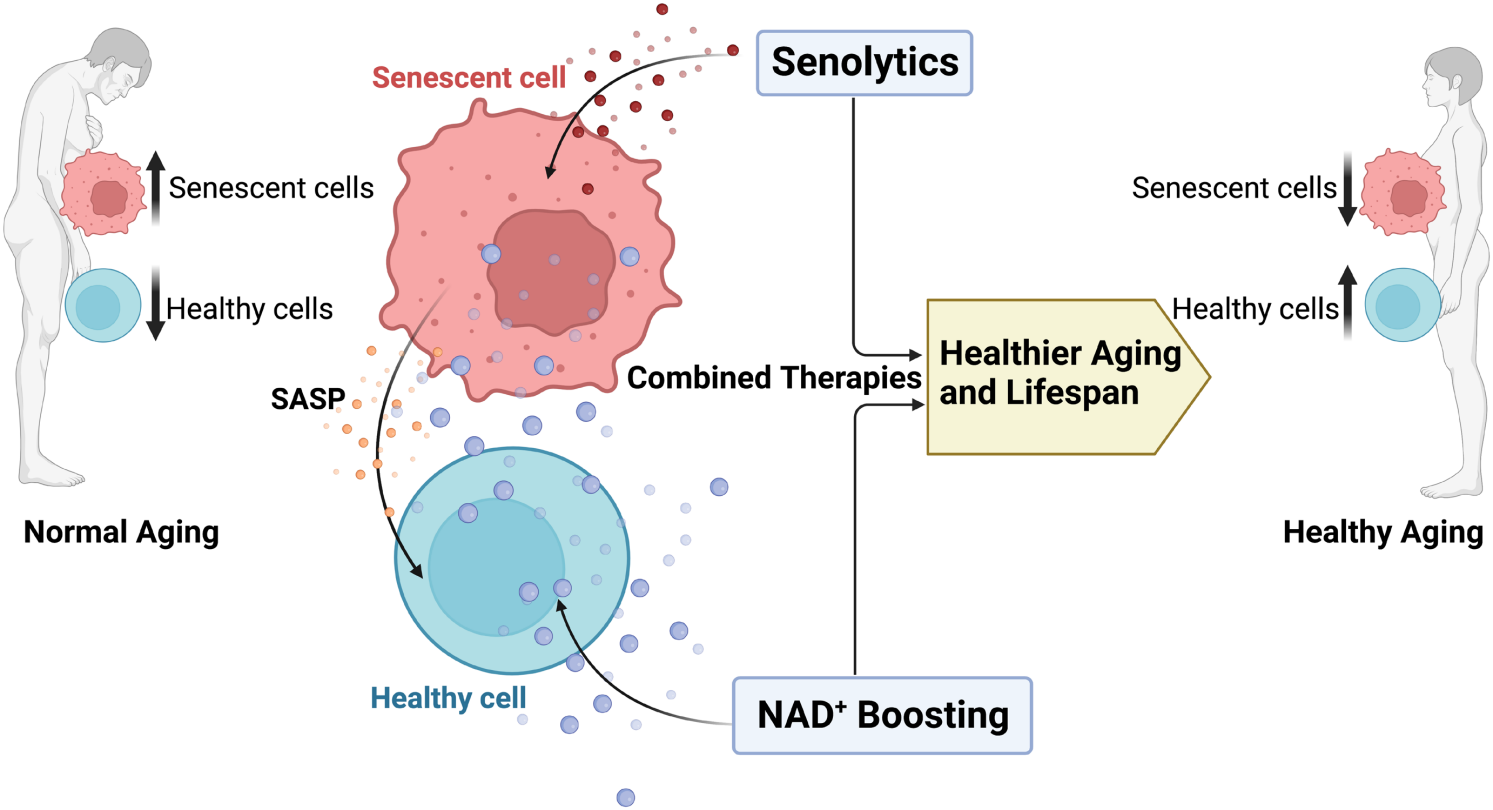
Theoretical framework for combined administration of senolytics and NAD^+^ boosting (replacement) therapies to promote healthier aging and lifespan. Senolytics selectively kill senescent cells and reduce overall senescence burden while NAD^+^ boosting globally supplements NAD precursors to different cell populations. Possibly, by combining these two therapies, the relative ratio of senescent cell to healthy cells would be reduced, the overall NAD^+^ level restored, leading to healthier aging, and increasing lifespan. NAD^+^, nicotinamide adenine dinucleotide; SASP, senescence‐associated secretory phenotype.

To expand our understanding of NAD metabolism and to safely employ NAD‐boosting therapies for aging and age‐related diseases, it is essential to determine which biological hallmarks of aging and phenotypes are ameliorated or exacerbated by NAD^+^ recovery (boosting) after a period of NAD^+^ decline. The effect of NAD^+^ boosting on biological hallmarks of aging, such as cellular senescence, and its potential deleterious effect on cancer progression have been extensively debated (Chini et al., 2021; Wiley & Campisi, 2021; Yaku et al., 2018). In particular, the effect of NAD^+^ recovery/boosting on the development and progression of cellular senescence needs to be further investigated in different cell types, tissues, and animal models. One possibility is that the potential benefits of NAD^+^ boosting therapy may be offset by the fact that this therapy may not decrease the senescence burden and may even exacerbate the SASP. Understanding the connection between NAD metabolism and senescence during the aging process would provide crucial knowledge to help the development of therapies for improving healthspan and preventing and treating age‐related diseases. Therefore, combining senolytic therapy with NAD boosting may enhance the efficacy and safety of NAD‐targeted interventions (Figure 4). Possibly, the combination of these therapies may have added/synergistic benefits in aging, healthspan, and longevity. However, to date no such studies have been reported. We believe that understanding the interplay between NAD metabolism and cellular senescence could greatly impact our understanding of the potential therapies for age‐related conditions. Thus, exploring the beneficial and adverse effects of combining NAD boosters with senolytic agents is imperative.

## AUTHOR CONTRIBUTIONS

CCSC and HSC participated in the design, writing, and editing. NLT and CCSC developed the figures. ENC participated in writing and editing.

## FUNDING INFORMATION

National Institutes of Health, Grant/Award Number: AG‐26094, AG58812, and CA233790. CTSA Grant Number TL1 TR002380 to NT from the National Center for Advancing Translational Science (NCATS).

## CONFLICT OF INTEREST STATEMENT

ENC holds a patent on CD38 inhibitors licensed by Elysium Health. ENC consults for Calico, Mitobridge, and Cytokinetics. Others declare no conflicts.

## Data Availability

N/A.
